# Changes in metabolite profiles caused by genetically determined obesity in mice

**DOI:** 10.1007/s11306-013-0590-1

**Published:** 2013-10-19

**Authors:** Nadine Schäfer, Zhonghao Yu, Asja Wagener, Marion K. Millrose, Monika Reissmann, Ralf Bortfeldt, Christoph Dieterich, Jerzy Adamski, Rui Wang-Sattler, Thomas Illig, Gudrun A. Brockmann

**Affiliations:** 1 Breeding Biology and Molecular Genetics, Department for Crop and Animal Sciences, Humboldt-Universität zu Berlin, Invalidenstr. 42, 10115 Berlin, Germany; 2 Research Unit of Molecular Epidemiology, Helmholtz-Zentrum München (GmbH), German Research Center for Environmental Health, Ingolstädter Landstr. 1, 85764 Munich/Neuherberg, Germany; 3 Berlin Institute for Medical Systems Biology at the Max-Delbrueck-Center for Molecular Medicine, Robert-Roessle-Str. 10, 13125 Berlin, Germany; 4 Institute of Experimental Genetics, Genome Analysis Center, Helmholtz Zentrum München, Ingolstädter Landstr. 1, 85764 Neuherberg, Germany; 5 Present Address: The Institute for Research in Operative Medicine, Faculty of Health, Department of Medicine, Witten/Herdecke University, Ostmerheimer Str. 200, 51109 Cologne, Germany; 6 Present Address: Hannover Unified Biobank, Medical School Hannover, Carl-Neuberg-Str. 1, 30625 Hannover, Germany

**Keywords:** Adiposity, Metabolism, Phosphatidylcholine

## Abstract

The Berlin Fat Mouse Inbred (BFMI) line harbors a major recessive gene defect on chromosome 3 (*jobes1*) leading to juvenile obesity and metabolic syndrome. The present study aimed at the identification of metabolites that might be linked to recessively acting genes in the obesity locus. Firstly, serum metabolites were analyzed between obese BFMI and lean B6 and BFMI × B6 F_1_ mice to identify metabolites that are different. In a second step, a metabolite–protein network analysis was performed linking metabolites typical for BFMI mice with genes of the *jobes1* region. The levels of 22 diacyl-phosphatidylcholines (PC aa), two lyso-PC and three carnitines were found to be significantly lower in obese mice compared with lean mice, while serine, glycine, arginine and hydroxysphingomyelin were higher for the same comparison. The network analysis identified PC aa C42:1 as functionally linked with the genes *Ccna2* and *Trpc3* via the enzymes choline kinase alpha and phospholipase A2 group 1B (PLA2G1B), respectively. Gene expression analysis revealed elevated *Ccna2* expression in adipose tissue of BFMI mice. Furthermore, unique mutations were found in the *Ccna2* promoter of BFMI mice which are located in binding sites for transcription factors or micro RNAs and could cause differential *Ccna2* mRNA levels between BFMI and B6 mice. Increased expression of *Ccna2* was consistent with higher mitotic activity of adipose tissue in BFMI mice. Therefore, we suggest a higher demand for PC necessary for adipose tissue growth and remodeling. This study highlights the relationship between metabolite profiles and the underlying genetics of obesity in the BFMI line.

**Electronic supplementary material:**

The online version of this article (doi:10.1007/s11306-013-0590-1) contains supplementary material, which is available to authorized users.

## Introduction

Obesity results from a sedentary lifestyle with malnutrition and low physical activity and is a risk factor for the development of the metabolic syndrome. The increasing number of obese humans correlates with the incidence of diabetes mellitus and coronary heart diseases (Kopelman 2000). Therefore, biomarkers for the diagnosis of metabolic dysfunctions would be desirable to initiate prevention or therapeutic programs early in life. A new approach for biomarker identification is the metabolomics (Shaham et al. 2008; Shah et al. 2010; Wang-Sattler et al. 2012).

As an emerging technology, metabolic profiling comprises the identification and quantification of molecules with a small molecular weight in biological fluids (Nicholson et al. 1999). Metabolites are influenced by factors such as age, sex and diet but also diseases such as diabetes (Williams et al. 2005; Lutz et al. 2006; Kim et al. 2011; Mittelstrass et al. 2011; Yu et al. 2012; Wang-Sattler et al. 2012). In mice, studies suggested metabolic profiling to be a surrogate for detecting dietary-induced insulin resistance or diabetes (Shearer et al. 2008). For example, previously, *N*-acetyl-l-leucine was identified as a potential biomarker for diabetes (Tsutsui et al. 2011). In humans, metabolic profiling was used to find alteration of plasma lipids to differentiate between healthy and diabetic subjects (Wang et al. 2005). In addition, three lyso-phosphatidylcholines (lysoPC) were reported as potential biomarkers for overweight in humans (Kim et al. 2010). Some studies associated metabolomics and transcriptomics to uncover the relationship between metabolites and gene expressions in different species (Askenazi et al. 2003; Bono et al. 2003; Urbanczyk-Wochniak et al. 2003; Hirai et al. 2004). Albeit several epidemiological studies and genome-wide association studies have repeatedly demonstrated that the development of obesity also depends on the genetic predispositions (Stunkard et al. 1986; Allison et al. 1996; Barsh et al. 2000; Speliotes et al. 2010), most metabolomic studies have not considered the underlying genetics.

Genetically well-defined mouse inbred models for obesity can contribute to identify metabolic markers for obesity and to link them to genetic determinants. We examined the Berlin Fat Mouse Inbred (BFMI) line which develops juvenile obesity (Wagener et al. 2006). Young BFMI mice are hyperphagic and develop the metabolic syndrome with impaired insulin sensitivity which was more pronounced on a high fat diet (HFD), and high serum triglyceride levels (Meyer et al. 2009; Hantschel et al. 2011). A region on chromosome 3, named *jObes1*, showed strong recessive gene effects on the obesity phenotype in a linkage study in the cross BFMI × C57BL/6NCrl (B6) (Neuschl et al. 2010).

The aim of the present study was the identification of candidate genes which are responsible for metabolite profiles associated with obesity in the obese BFMI mouse model. We have analyzed metabolites in BFMI mice in comparison with lean B6 mice and the F_1_ offspring of the cross BFMI × B6 which are lean as well due to the recessive *jobes1* effect. A metabolite–protein network analysis was performed connecting significantly differentially regulated metabolites with candidate genes for obesity of the *jObes1* region on mouse chromosome 3.

## Materials and methods

### Animals and diets

In this study we used the lines BFMI860/Hber (BFMI) and C57BL/6NCrl (B6) and F_1_ individuals generated by crossing BFMI and B6 mice. A detailed description of the breeding history of the BFMI line is outlined in Wagener et al. (2006). In brief, the BFMI line was generated from the outbred population Berlin Fat Mouse (BFM). Founders of BFM mice were originally purchased from pet shops and subsequently selected first for low protein content, second for low body mass and high fat content and then for high fatness for 58 generations before inbreeding. As no control line of the selection experiment exists, we used B6 mice of the substrain C57BL6/NCrl as lean controls (Charles River Laboratories, Sulzfeld, Germany) which were also used to map genetic loci affecting obesity in the cross BFMI × B6 (Neuschl et al. 2010). Mice were reproduced in our animal facility at the Humboldt-Universität zu Berlin. Mice were kept at room temperature (22–24 °C) with a light dark cycle of 12 h. After weaning at the age of 3 weeks, 4–5 mice of each line (BFMI, B6 and F_1_) and of each sex were randomly chosen and placed on either a standard maintenance diet (SMD) containing 12.8 MJ/kg metabolizable energy with 9 % of its energy from fat, 33 % from protein content and 58 % from carbohydrates (V1534-000 ssniff R/M-H, ssniff Spezialdiäten GmbH, Soest, Germany) or a HFD containing 19.1 MJ/kg metabolizable energy with 45 % of its energy from fat, 24 % from protein content and 31 % from carbohydrates (S8074-E010 ssniff EF R/M, ssniff Spezialdiäten GmbH, Soest, Germany). The standard diet derived its fat from soy oil, whereas the high-fat diet derived its fat from coconut oil and suet. The animals had ad libitum access to diets and water. All animal treatments were in accordance to the German Animal Welfare Legislation (approval no. G0152/04, T0149/04).

### Body weight, body composition, blood collection and the measurement of serum parameters

At the age of 10 weeks body weight and body fat mass were determined by a quantitative magnetic resonance analysis using the EchoMRI whole body composition analyzer (Echo Medical Systems, Houston, Texas, USA) (Taicher et al. 2003; Tinsley et al. 2004). The recorded fat mass represented the total fat mass in the body.

After a fasting period of 2 h and anesthesia with isofluran, 10 weeks old mice were sacrificed and reproductive adipose tissue, liver, brain, pancreas and blood was collected. Serum was recovered by centrifugation for 15 min at 600×*g*. Serum and tissues were stored at −80 °C until analysis.

Serum triglycerides were determined using the Fluitest TG (Analyticon Biotechnologies AG, Lichtenfels, Germany). Serum non-esterifies free fatty acids (NEFAs) were measured using the NEFA-HR(2) kit (Wako Chemicals GmbH, Neuss, Germany).

### Measurement of metabolites

For the measurement of metabolites a 10 μl volume of serum samples was used. The Hamilton Star robotics (Bonaduz, Switzerland) was used for liquid handling of samples. A total of 163 metabolite molecules including 41 acylcarnitines, 14 amino acids, one sugar, 92 glycerophospholipids and 15 sphingolipids were measured (Table S1) using the Absolute*IDQ* ^TM^ p150 Kit following the manufacturer’s instructions (BIOCRATES Life Sciences AG, Innsbruck, Austria) on the ion trap mass spectrometry API 4000 Q TRAP LC/MS/MS System (Applied Biosystems Deutschland GmbH, Darmstadt, Germany). The Absolute*IDQ* ^TM^ p150 kit has been described in detail previously (Illig et al. 2010; Römisch-Margl et al. 2011). Analytics were calculated in μmol concentrations using the Met*IQ* software which was integrated in the Absolute*IDQ* ^TM^ kit. To control the quality of metabolites, the coefficients of variation (CV) were calculated using the reference samples for each metabolite and 43 metabolites with CV higher than 0.2 were excluded and 120 metabolites were remained for further analysis (Mittelstrass et al. 2011; Wang-Sattler et al. 2012).

### Statistics

#### ANOVA of phenotypes and metabolites

Phenotypic data were analyzed by performing the analysis of variance (ANOVA) to assess the effect of lines using the SAS version 9.1.3 (SAS Institute Inc., Cary, NC, USA). Multiple pairwise comparisons were Bonferroni-corrected. Gene expression data analysis was performed using a two tailed student’s *t*-test (GraphPad Prism 5 Software, San Diego, CA/USA). Differences were considered statistically significant at *p* < 0.05.

The remaining 120 out of 163 metabolites were log-transformed to remove skewness. In a first analysis the effects of sex, diet, and line on metabolite levels of BFMI, B6, and F_1_ mice were assessed by ANOVA. To further test the recessive allele effect associated with obesity in BFMI mice, B6 and F_1_ mice were combined to one group which was compared with BFMI mice in a second analysis. B6 and F_1_ mice were both lean and did not differ in fat deposition.

Metabolites differing significantly between BFMI mice and the group of B6 and F_1_ mice, that showed the expected recessive allele effect, were selected for further analyses.

### Random forest, stepwise selection methods and candidate metabolite selection

To select candidate metabolites that are linked to genetic differences between obese and lean mice, we applied two more methods, the non-parametric random forest (Breiman 2001) and the parametric stepwise selection, which assessed the metabolites as a group. The supervised classification method random forest was used to select metabolites of importance among the 31 highest ranking variables between the two groups of obese and lean mice. Those 31 metabolites showed most impact on obesity in the internal permutation test of random forest. Furthermore, selected metabolites were used for a stepwise selection method on the logistic regression model. Here, metabolites were used which showed both significantly different concentrations between the two compared groups in the logistic regression and which were also selected using random forest. Those metabolites were added and dropped from the model one by one. Akaike’s Information Criterion (AIC) was used to evaluate the performance of the subsets of metabolites used in the models. The model with minimal AIC was finally chosen and metabolites left in this model were potential independent metabolites that best distinguish lean from obese mice. Correlated metabolites with less separation power were dropped. The area under the receiver-operating-characteristic curves (ROC) was used to evaluate the models and a likelihood ratio test was used to compare the models. Calculations were performed under the R statistical environment (R Core Team 2013).

#### Network analysis

Metabolite–protein interactions from the Human Metabolome Database (HMDB) (Wishart et al. 2009) and protein–protein interactions in the Search Tool for the Retrieval of Interacting Genes/Proteins (STRING) (Jensen et al. 2009) were used to construct a network containing relationships between metabolites, enzymes and obesity-related genes (He et al. 2012; Xu et al. 2013). The candidate metabolites were assigned to the HMDB IDs using the metaP-Server (Kastenmuller et al. 2011). Their associated enzymes were derived according to the annotations provided by HMDB. We chose six obesity candidate genes of the *jObes1* region on chromosome 3 to connect metabolites through the associated enzymes. In the search for links, we allowed an intermediate protein through STRING and optimization by eliminating edges with a STRING score below 0.7 and undirected paths. The sub-networks were connected by the shortest path from metabolites to obesity candidate genes.

### Gene expression analysis and sequencing

Gene expression analyses were performed with SMD-fed male mice of BFMI, B6 and F1 (n = 5–9 per group). Total RNA was isolated from liver, brain and pancreas using the nucleic acid and protein purification kit (Machery-Nagel, Düren, Germany) following the suppliers protocol, and from reproductive adipose tissue by acid guanidinium thiocyanate–phenol–chloroform extraction. Genomic DNA was removed using the Turbo DNA-*free* ^TM^ Kit (Applied Biosystems, Foster City CA/USA; Ambion, Austin TX/USA). 1 μg RNA was reverse-transcribed into cDNA using AccuScript^®^ High Fidelity Transcriptase and oligo-dT primers according to the manufacturer’s instructions (Stratagene Europe, Agilent Technologies, Waldbronn, Germany). Primers for quantitative real-time PCR were designed using Primer3 software (http://frodo.wi.mit.edu/) and are given in Table S4. Primers were checked with a 20 μl control PCR contained 50 ng cDNA, 10 μM of each primer pair and 1 Unit FIRE-Pol^®^ DNA Polymerase (Solis BioDyne, Tartu, Estonia) with a three step PCR standard program. Fragment length of PCR products were checked on an agarose gel. Quantitative real-time PCR was performed in a total reaction volume of 10 μl containing MasterMix Plus for SYBR^®^ Assay (Eurogentec, Cologne, Germany), 10 ng cDNA and 10 μM of the gene specific primers. Triplicates were measured on Viia^TM^ 7 Real-Time PCR System (Applied Biosystems, Darmstadt, Germany). Gene expression was calculated as relative quantity (RQ) using the ΔΔCt method (Livak and Schmittgen 2001). As endogenous controls, *Rps25* and *b*-*Actin* were chosen and gene expression was calculated relative to the group of B6 and F_1_ mice, normalized to a value of 1.

The coding region and 420 bp upstream of the first exon of *Ccna2* were sequenced using cDNA and genomic DNA of BFMI and B6 mice, respectively. Genomic DNA was extracted from spleen using phenol and chloroform in a standard procedure. Sequencing primers were designed with DNASTAR software (DNASTAR Inc., Madison, USA) and are given in Table S4. PCR products, amplified using standard methods, were cut from a 2.0 % agarose gel and purified using GeneJETTM Gel Extraction Kit (Fermentas, St. Leon-Rot, Germany). The sequence reactions in both directions were performed with BigDye^®^ Terminator v1.1 Ready Reaction Cycle Sequencing Kit and an ABI PRISM^®^ 310 Genetic Analyser (Applied Biosystems, Darmstadt, Germany) following manufacturers instruction. Sequences were assembled and analyzed using the DNASTAR software (DNASTAR Inc., Madison, USA). The chromosome position of found variations and reference alleles are based on Ensembl release 67—May 2012, Mouse (NCBIM37). Transcription factor binding sites were determined with the web tool CONSITE (Sandelin et al. 2004) using human and mouse transcription factor model matrices and a scoring threshold of 80 %. The 3′ UTR of the *Ccna2* reference transcript (NM_009828) was scanned for binding sites of known mouse miRNAs using the web program PITA (Kertesz et al. 2007) with standard parameters. Resulting energetic scores estimate the free binding energy in the seed region of the miRNA–mRNA duplex and thus the binding strength of the miRNA to the given 3′UTR site. Only scores equal or below −10 were considered as these are likely to be functional in endogenous miRNA expression levels (Kertesz et al. 2007). Expression data of transcription factors were taken from the arrays GeneAtlas MOE430 and GNF1M via the web tool BioGPS (http://biogps.org). MicroRNA expression data were obtained from the Gene Expression Atlas (http://www.ebi.ac.uk/gxa/).

## Results and discussion

### Phenotypic and metabolic characteristics of BFMI, B6 and F_1_ mice

Ten weeks old BFMI males on standard maintenance diet (SMD) had a 4.1 and 4.6 and females a 3.0 and 2.2 times higher body fat content compared to male and female B6 and F_1_ mice (*p* < 0.001) respectively (Table 1). Feeding a high-fat diet (HFD), all mice gained additional fat mass. Due to the recessive *jObes1* effect in BFMI mice leading to obesity (Neuschl et al. 2010) BFMI males showed 3.1- and 2.2-fold and females 3.0- and 2.8-fold higher body fat content at 10 weeks compared with its B6 and F_1_ counterparts (*p* < 0.001), respectively (Table 1). In addition, serum concentrations of NEFAs were about 1.4 times elevated (*p* < 0.01) in both sexes of BFMI mice fed an SMD compared with lean F_1_ and B6 mice. The differences were lower in HFD-fed mice with only 1.05 and 1.25 times increased NEFAs in BFMI males and females, respectively. In accordance to the recessive *jObes1* effect, F_1_ and B6 males showed no statistical difference in their body fat content and NEFA serum levels, neither on SMD nor on HFD (Table 1). In contrast, serum triglyceride levels of BFMI mice were about two times as high as in B6 mice on both diets and both sexes (*p* < 0.001), but they showed no difference to lean F_1_ animals which had also high triglyceride serum levels.

**Table 1:** Phenotypic characteristics of BFMI, B6, and BFMI × B6 F_1_ mice

Trait	BFMI				B6				F1				ANOVA effects of		
	Males		Females		Males		Females		Males		Females		Line	Sex	Diet
	SMD	HFD	SMD	HFD	SMD	HFD	SMD	HFD	SMD	HFD	SMD	HFD			
BW	38.3	48.5	27.3	38.6	26.5	25.7	21.5	22.8	28.4	36.2	25	25.4	<0.001	<0.001	<0.001
(g)	(1.6)	(3.8)	(1.5)	(6.5)	(1.2)	(1.4)	(0.7)	(2.4)	(0.8)	(2.3)	(1)	(0.7)			
FAT	23.89	31.91	20.79	38.53	5.87	10.35	6.87	12.65	5.14	14.31	9.42	13.63	<0.001	n.s.	<0.001
(%)	(3.49)	(1.51)	(4.15)	(3.88)	(2.119)	(4.24)	(2.37)	(5.71)	(2.25)	(3.27)	(1.22)	(4.27)			
TG	190.3	151.5	103.1	127.7	102.4	68.9	59.2	69.8	169.5	131.7	148.4	73.2	<0.001	<0.001	0.017
(mg/dl)	41.2	35.6	28	74	34.9	28.8	21	11.7	43.2	27.3	22	16.9			
NEFA	1	1	0.8	1	0.7	0.8	0.7	0.7	0.7	0.8	0.8	0.8	0.001	n.s.	n.s.
(mmol/l)	0.1	0.1	0.2	0.3	0.1	0.1	0.2	0.1	0.1	0.1	0.2	0.1			

Values represent means and standard deviations in parentheses. ANOVA results were considered as statistically significant at *p* < 0.05

For the determination of metabolite profiles, metabolites were first compared between the high-fatness selected obese BFMI line with lean B6 and BFMI × B6 F_1_ mice in both sexes and on two diets. Out of 163 targeted metabolites 120 were above the detection limit and passed the quality control and, therefore, were used in this study; 14 metabolites differed between BFMI and B6 or between BFMI and F_1_ mice (Table S2). B6 and F_1_ mice did not differ in their metabolite concentrations indicating that the recessive genetic defect of BFMI mice is mainly responsible for metabolic differences when comparing lean and BFMI mice. Although sex differences in the metabolic profile had been reported in humans (Lutz et al. 2006; Mittelstrass et al. 2011), ANOVA provided evidence that the sex had no effect in this study. Furthermore, also the diet had almost no effect, except for diacyl-phosphatidylcholine (PC aa) C42:2 which was affected by diet, but not by line (Table S2). Therefore, the metabolite PC aa C42:2 as well as the factors sex and diet were not considered in subsequent analyses. As the focus of this study was on differences in metabolite concentrations due to genetic variations between obese BFMI and lean B6 and F_1_ mice, all BFMI mice of the SMD and HFD groups were combined to one group of obese mice whereas all B6 and F_1_ mice were combined to a group of lean mice. This is consistent with our expectation of the recessive mode of action of the obesity effect of the BFMI line leading to juvenile obesity. Due to the increase of group sizes further 18 metabolites were identified to be different between obese BFMI and the lean mice in addition to the 13 metabolites that differed between BFMI and either B6 or F_1_ mice (Table 2). Overall, BFMI mice had a reduction in 12 serum PC aa, 10 PC ae, 2 lysoPC, 3 acylcarnitines and increased levels of 3 amino acids and 1 sphingomyeline when comparing to lean mice. Because these 31 metabolites differed between obese BFMI and lean B6 and F_1_ mice, we suggest that at least one gene of the genomic chromosome 3 region, responsible for obesity in BFMI mice, accounts for the amount of at least one metabolite in serum of the mice.

**Table 2:** Metabolites that differ significantly between obese BFMI and the group of lean B6 and F_1_ mice

Metabolite short name	Metabolite biochemical name	BFMI	B6 + F_1_	ANOVA effect of line
PC aa C34:1	Phosphatidylcholine diacyl C34:1	213.02 ± 124.9	313.65 ± 188.48	0.042
PC aa C36:3	Phosphatidylcholine diacyl C36:3	136.34 ± 55.02	181.21 ± 73.58	0.017
PC aa C36:4	Phosphatidylcholine diacyl C36:4	169.75 ± 47.87	202.41 ± 68.46	0.049
PC aa C38:4	Phosphatidylcholine diacyl C38:4	135.81 ± 66.55	181.75 ± 86.61	0.045
PC aa C38:5	Phosphatidylcholine diacyl C38:5	66.1 ± 42.79	98 ± 54.63	0.016
PC aa C40:2	Phosphatidylcholine diacyl C40:2	0.48 ± 0.16	0.59 ± 0.2	0.014
PC aa C40:3	Phosphatidylcholine diacyl C40:3	0.78 ± 0.45	1 ± 0.47	0.033
PC aa C40:4	Phosphatidylcholine diacyl C40:4	3.31 ± 1.71	4.52 ± 2.28	0.028
PC aa C40:5	Phosphatidylcholine diacyl C40:5	14.83 ± 13.34	23.34 ± 16.81	0.05
PC aa C42:1	Phosphatidylcholine diacyl C42:1	0.15 ± 0.04	0.17 ± 0.04	0.005
PC aa C42:5	Phosphatidylcholine diacyl C42:5	0.34 ± 0.12	0.4 ± 0.1	0.02
PC aa C42:6	Phosphatidylcholine diacyl C42:6	0.83 ± 0.23	1 ± 0.3	0.02
PC ae C36:0	Phosphatidylcholine acyl-alkyl C36:0	0.92 ± 0.55	1.18 ± 0.59	0.048
PC ae C38:1	Phosphatidylcholine acyl-alkyl C38:1	2.35 ± 1.87	3.62 ± 2.49	0.05
PC ae C38:3	Phosphatidylcholine acyl-alkyl C38:3	3.51 ± 2.18	4.86 ± 2.7	0.049
PC ae C40:4	Phosphatidylcholine acyl-alkyl C40:4	3.19 ± 1.36	4 ± 1.57	0.036
PC ae C40:5	Phosphatidylcholine acyl-alkyl C40:5	2.2 ± 1.33	2.94 ± 1.52	0.036
PC ae C42:0	Phosphatidylcholine acyl-alkyl C42:0	1.03 ± 0.3	1.29 ± 0.45	0.017
PC ae C42:1	Phosphatidylcholine acyl-alkyl C42:1	0.72 ± 0.18	0.92 ± 0.35	0.008
PC ae C42:2	Phosphatidylcholine acyl-alkyl C42:2	0.83 ± 0.53	1.17 ± 0.7	0.046
PC ae C44:3	Phosphatidylcholine acyl-alkyl C44:3	0.13 ± 0.03	0.16 ± 0.04	0.006
PC ae C44:4	Phosphatidylcholine acyl-alkyl C44:4	0.16 ± 0.07	0.18 ± 0.05	0.015
lysoPC a C16:1	lysoPhosphatidylcholine acyl C16:1	10.5 ± 3.68	12.69 ± 3.92	0.049
lysoPC a C18:1	lysoPhosphatidylcholine acyl C18:1	90.23 ± 58.24	121.75 ± 62.9	0.048
Ser	Serine	108.43 ± 47.37	81.63 ± 31.65	0.009
Gly	Glycine	232.3 ± 59.7	194.3 ± 59.9	0.025
Arg	Arginine	152.8 ± 38.5	132.2 ± 39.2	0.031
C14	Tetradecanoylcarnitine	0.08 ± 0.02	0.1 ± 0.05	0.003
C14:1	Tetradecanoylcarnitine	0.12 ± 0.03	0.14 ± 0.03	0.003
C18:1	Octadecanoylcarnitine	0.15 ± 0.05	0.21 ± 0.09	0.002
SM (OH) C22:1	Hydroxysphingomyelin C22:1	4.19 ± 2.75	3.16 ± 1.56	0.04

Values are shown as means ± standard deviations. Differences were considered statistically significant at *p* < 0.05

Most metabolites that differed between obese and lean mice belonged to the group of glycerophospholipids that were all significantly lower in BFMI mice (*p* < 0.05). The glycerophospholipid PC aa C42:1 showed highest significance (*p* = 0.005). The amino acids serine, glycine and arginine and one hydroxyshingomyelin C22:1 (SM (OH) C22:1) were higher in BFMI mice.

### Selection of candidate metabolites

**Fig. 1:**
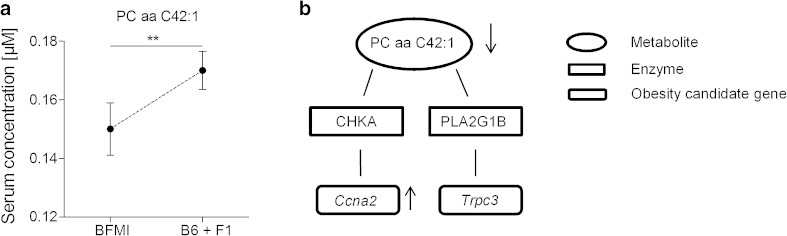
Effects of the metabolite PC aa C42:1. **a** Serum concentration of the metabolite PC aa C42:1 in BFMI and the combined group of B6 and F1 mice are given in mean ± standard error. **b** Network composed of PC aa C42:1, the interacting enzymes CHKA and PLA2G1B and the underlying genes *Ccna2* and *Trpc3*. *CHKA* choline kinase α; *PLA2G1B* phospholipase A2 group 1B; *Ccna2* cyclin A2 and *Trpc3* transient receptor potential cation channel subfamily C, member 3

Using the 31 metabolites that were found to be significantly different between lean and obese mice, the random forest analysis identified the acylcarnitine C14, the amino acid serine and PC aa C42:1 with high confidential ROC curve AUC-scores of 0.752, 0.711 and 0.725, respectively, as those metabolites that were mainly affected by the genetic background and explained best the genetic differences between lean and obese mice. Serum serine level was increased in the obese line compared with lean mice, while the amount of acylcarnitine C14 and PC aa C42:1 was reduced in BFMI mice. To find the obesity candidate genes which might be involved in the regulation of metabolites, the selected candidate metabolites were connected to obesity candidate genes of the chromosome 3 *jObes1* region by a network analyses. Out of the three significant metabolites, only PC aa C42:1 showed a functional link to the obesity candidate genes of the *jObes1* locus. In detail, PC aa C42:1 showed a connection to the genes encoding for cyclin A2 (*Ccna2*) and the transient receptor potential cation channel subfamily C, member 3 (*Trpc3*) (Fig. 1) of the *jObes1* locus. The interaction is given via the enzymes choline kinase alpha (CHKA) and PLA2G1B, respectively.

### Obesity candidate gene characterization

**Fig. 2:**
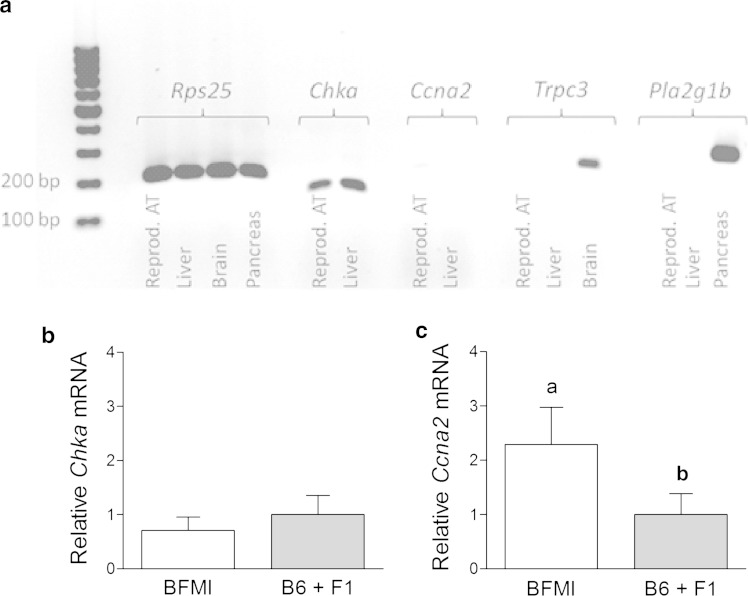
Gene expression of candidate genes of the network analysis in obese BFMI and lean B6 and F1 mice. **a** PCR products of *Rps25*, *Chka*, *Ccna2*, *Trpc3* and *Pla2g1b* in reproductive adipose, liver, brain and pancreas tissue of BFMI mice. **b** Relative mRNA expression level of *Chka* in reproductive adipose tissue (n = 5–9). **c** Relative mRNA expression level of *Ccna2* in reproductive adipose tissue (n = 5–9). Bar graphs are given as mean values plus standard deviation. Bar graphs with different letters are significantly different at *p* < 0.05

To characterize the potential impact of the obesity candidate genes on the lower PC aa C42:1 serum metabolite level in BFMI mice, gene expression analyses of the network genes *Ccna2*, *Chka*, *Pla2g1b* and *Trpc3* were performed. Although weakly expressed, the obesity candidate gene *Ccna2* was 2.3-times up-regulated in BFMI compared with B6 and F1 mice in the reproductive adipose tissue (Fig. 2). However, the adipogenic mRNA amount of *Chka* encoding the interacting enzyme did not differ between BFMI and B6 mice. In the liver, only *Chka* was found to be expressed, whereas *Ccna2* mRNA was not detected. The hepatic *Chka* expression did not differ between groups. *Pla2g1b* and *Trpc3* were expressed neither in the reproductive adipose tissue nor in the liver, but could be detected in the pancreatic and brain tissue, respectively. However, as only *Ccna2* and *Chka* were expressed in adipose tissue which is one of the main sites for metabolite metabolism, only *Ccna2* seems to be the obesity gene which contributes to reduced serum phosphatidylcholine concentrations in BFMI mice.

To further characterize the different expressions of *Ccna2* between BFMI and B6 mice, comparative sequencing of the promoter and coding regions was performed. Overall, 29 mutations were found when comparing the sequences, thereof 24 SNPs and five InDels. One deletion in the promoter region, two SNPs in the 5′ untranslated region (UTR), one coding SNP in exon 1, one SNP and three InDels in the 3′UTR were unique to the BFMI line compared with the reference sequence of B6 and the SNP database (Welcome Trust Sanger Institute, http://www.sanger.ac.uk/cgi-bin/modelorgs/mousegenomes/snps.pl) (Table 3). The two SNPs in the protein coding region were synonymous. The in silico analysis of the detected variants revealed seven SNPs and one InDel in the sequenced 420 bp promoter region as well as two SNPs in the 5′-UTR/exon1 region that are predicted to gain or lose transcription factor binding sites or modify the binding capacity in BFMI mice compared with B6 (Table 3). Furthermore, seven SNPs and two insertions create new micro RNA recognition sites and one SNP, one deletion and the two insertions lead to the loss of micro RNA binding sites in the 3′ UTR (Table 3). Since some transcription factors and micro RNAs occur in the adipose tissue (Table S3), both variations in the promoter and the 3′ UTR could affect the amount of *Ccna2* transcripts in the adipose tissue.

**Table 3:** Variations in the *Ccna2* promoter and coding region between BFMI and B6 (reference) mice

Variation-ID	Position	B6 allele	BFMI allele	rsID	Genomic region	Function	Effect in BFMI relative to B6	Factor^a^
SNP1	36471433	G	A	rs49416676	Promoter	TF-regulation	gain silencing	RORA c-FOS
SNP2	36471338	G	A	rs50741947	Promoter	TF-regulation	gain loss enhancing	NKX (NKX2-3), SOX17, SOX9, FREAC7 (FOXL1) SPI-B, FREAC3 (FOXC1) FREAC2 (FOXF2), SRY, SOX5, FREAC4 (FOXD1)
SNP3	36471316	A	G	rs46760941	Promoter	TF-regulation	loss silencing	FREAC7 (FOXL1), GATA2, GATA3, MZF_1-4 (MZF1) Gklf, SPI-B
SNP4	36471291	A	G	rs50888702	Promoter	TF-regulation	loss enhancing silencing	Irf-1, SPI-1, SPI-B GATA-3 Gklf
SNP5	36471264	G	A	rs48075421	Promoter	TF-regulation	gain loss enhancing silencing	Gklf SP1 Pax-2 TFAP2A
SNP6	36471206	A	G	rs45964814	Promoter	TF-regulation	gain enhancing	TFAP2A, MZF1_5-13 (MZF1), MZF1_1-4 (MZF1) Gklf (Klf4), SPI-1, SPI-B
SNP7	36471126	C	G	rs51039343	Promoter	TF-regulation	loss enhancing unchanged	Elk-1 SP1, TFAP2A SPI-1
DEL1	36471074	A	–	novel	Promoter	TF-regulation	gain loss silencing	Hen1 (Nhlh1), Myf (Myog) Elk-1, TFAP2A SPI-1
SNP8	36471071	G	T	novel	5′UTR/Exon1	TF-regulation		
SNP9	36470786	T	C	novel	5′UTR/Exon1	TF-regulation	gain loss	TFAP2A SP1
SNP10	36470691	C	T	novel	Exon1/coding	synonymous coding	Leucin	YTG
SNP11	36467695	G	A	rs49497566	Exon4/coding	synonymous coding	Glutamin	CAR
SNP12	36465032	T	C	rs29940755	Exon8/3′UTR	unknown	–	–
SNP13	36464877	A	G	rs29939979	Exon8/3′UTR	miRNA-regulation	Gain	mmu-miR-539
SNP14	36464857	G	A	rs29939976	Exon8/3′UTR	miRNA-regulation	Gain	mmu-miR-669
SNP15	36464725	C	T	rs45880100	Exon8/3′UTR	unknown	–	–
INS1	36464688	–	T	rs29939053	Exon8/3′UTR	miRNA-regulation	Gain	mmu-miR-671
SNP16	36464653	G	C	rs29939050	Exon8/3′UTR	unknown	–	–
SNP17	36464611	A	T	rs29939047	Exon8/3′UTR	miRNA-regulation	Gain	mmu-miR-342, -705, -450, -762, -296
DEL2	36464525	ACAA	–	novel	Exon8/3′UTR	miRNA-regulation	Loss	mmu-miR-208
SNP18	36464511	A	G	rs29939044	Exon8/3′UTR	–	–	–
SNP19	36464492	C	T	rs29938061	Exon8/3′UTR	miRNA-regulation	Gain	mmu-miR-466
SNP20	36464477	A	G	novel	Exon8/3′UTR	miRNA-regulation	Gain	mmu-miR-466, -574, -362, -342, -467
INS2	36464469	–	G	novel	Exon8/3′UTR	miRNA-regulation	gain loss	mmu-miR-466, -467, -297, -669, mmu-miR-297, -669
INS3	36464464	–	GTGTATATACATACACACACATATACAC	novel	Exon8/3′UTR	miRNA-regulation	enhancing gain loss	mmu-miR-466, -467, -297 mmu-miR-466 mmu-miR-669f
SNP21	36464447	G	C	rs47451785	Exon8/3′UTR	–		
SNP22	36464390	G	C	rs29938058	Exon8/3′UTR	miRNA-regulation	loss	mmu-miR-470, -330, -362, -211, -204
SNP23	36464274	C	T	rs29938055	Exon8/3′UTR	–	–	–
SNP24	36463910	A	G	rs29944107	Exon8/3′UTR	miRNA-regulation	gain	mmu-miR-759, -665, -433

The promoter region comprises 420 bp upstream of the first exon according to the transcript ENSMUST00000029270. This transcript is encoded in the minus strand; hence alleles are given according to the minus strand. The chromosomal position and reference alleles are based on Ensembl release 67, Mouse (NCBIM37). Sequence variants without reference ID are novel. *TF* transcription factor

^a^Numbers refer to mmu-miR-ID numbers of micro RNAs. Transcription factor binding sites were determined with the web tool CONSITE (Sandelin et al. 2004) using human and mouse transcription factor model matrices and a scoring threshold of 80 %. The 3′ UTR of the *Ccna2* reference transcript (NM_009828) was scanned for binding sites of known mouse miRNAs using the web-programme PITA (Kertesz et al. 2007) using standard parameter. Resulting energetic scores estimate the free binding energy in the seed region of the miRNA–mRNA duplex and thus the binding strength of the miRNA to the given 3′UTR site. Only scores equal or below −10 were considered as these are likely to be functional in endogenous miRNA expression levels (Kertesz et al. 2007)

### Metabolite pathways and the biological context

Generally, PCs account for 50 % of eukaryotic membrane phospholipids (van Meer et al. 2008) and are essential for coating lipid droplet surfaces (Krahmer et al. 2011). Further, PCs stimulate adipocyte differentiation and increase triglyceride levels of 3T3-L1 and preadipocytes (Zhang et al. 2009). Apart from structural functions of PCs in membranes, they serve as substrates for the synthesis of diacylglycerides and subsequent triglycerides and free fatty acids in the liver. In the BFMI line lowered levels of several serum PCs and higher triglyceride and free fatty acid synthesis compared with lean mice also indicate the important role of PCs in fat metabolism. Therefore, PC emerged as most interesting metabolite in connection with obesity, but a direct link between the specified PC aa C42:1 and obesity has not been found in the literature now.

Within the metabolite–protein network the interaction of PC aa 42:1 with the obesity candidate genes *Ccna2* and *Trpc3* was given via the enzymes CHKA and PLA2G1B, respectively. However, only *Ccna2* and *Chka* were expressed in adipose tissue which is one of the main sites for metabolite metabolism. Thus, *Ccna2* seemed to be the obesity gene which contributed to lower serum PC concentrations in BFMI mice. The functional role of the pathway including the enzyme CHKA and PC aa C42:1 became evident as CHKA catalyzes the initial phosphorylation step of choline within PC synthesis via the cytidine diphosphocholine pathway (Kent 2005). CCNA2 in turn is a cyclin family member, which is well known to regulate mitotic cell division by associating to cyclin dependent protein kinases (Johnson and Walker 1999). A gene disruption resulted in embryonic lethality (Murphy et al. 1997) demonstrating its essential regulatory role. An elevated expression of *Ccna2* in the reproductive adipose tissue of BFMI mice likely leads to increased mitotic activity of adipocytes. While there was no indication for an increased adipocyte number in BFMI mice (Wagener et al. 2010), the size of adipocytes was increased to compensate the higher lipid storage and an ongoing turnover of adipocytes can be assumed. This assumption is in line with human studies reporting on more newly generated adipocytes in obese adults than in lean adults, despite constant adipocyte numbers (Spalding et al. 2008).

An increased adipocyte turnover in BFMI mice required PCs as an essential membrane component. PC aa C42:1 seemed to be one of the major membrane PCs that may be regulated by *Ccna2* in the obese line. The demand for high PC amounts for membrane production was jointly responsible for lower available serum PCs in BFMI mice, in particular as cytidinediphosphocholine pathway seemed to be normal which was indicated by unchanged *Chka* expression between obese and lean mice.

Since no hepatic *Ccna2* expression could be detected in BFMI and B6 mice, we suggest a tissue-specific expression pattern which goes along with the specific increase of adipocyte production in obese subjects. The genetic modifications in the promoter region of *Ccna2* cause new or lost putative transcription factor binding sites in the BFMI line and might be responsible for differential regulation of expression between lean and obese mice. Furthermore, an increase in mRNA stability due to the comprehensive variations in the 3′ untranslated region cannot be excluded and could lead to increased translation ending in a higher CCNA2 protein availability within the adipose tissue of BFMI mice. Since several binding sites of transcription factors and miRNAs that occur in adipose tissues are modified, the transcriptional or translational regulation of the alternative *Ccna2* haplotype in BFMI is likely.

The second pathway branch linked *Trpc3* with PC aa C42:1 via PLA2G1B. *Trpc3* as a member of the transient receptor potential superfamily encodes for cation channels. Cations such as calcium are required for PLA2G1B action which is secreted by the pancreas into the intestinal lumen to digest dietary fatty acids (Carey et al. 1983). Since no gene expression of *Trpc3* was observed either in the reproductive adipose tissue or in the liver between BFMI and B6 mice, we assumed that this pathway is not involved in the regulation of PC aa C42:1.

## Concluding remarks

In the present study, serum metabolites of genetically obese BFMI and lean mice were compared. Secondly, significantly different metabolites were linked with underlying obesity candidate genes revealing PC aa C42:1 as being influenced by the genetic background for obesity in BFMI mice. *Ccna2* and *Trpc3* were selected as candidate genes affecting PC aa C42:1 serum levels via the enzymes CHKA and PLA2G1B. To uncover the biological context of the specified PC aa C42:1 and respective genes, expression analysis and comparative sequencing were performed in BFMI and lean mice. Unique mutations in the *Ccna2* promoter of obese mice were identified which are located either in transcription factor or micro RNA binding sites. These genetic modifications are postulated to affect *Ccna2* gene expression in adipose tissue likely leading to higher mitotic activity of adipocytes. In conclusion, adipose tissue growth and remodeling are increased in obese mice and cause a higher demand of specific PCs.

## Electronic supplementary material

Below is the link to the electronic supplementary material.

Supplementary material 1 (DOCX 25 kb)

Supplementary material 2 (DOCX 23 kb)

Supplementary material 3 (DOCX 28 kb)

Supplementary material 4 (DOCX 16 kb)
