# Supplementary material for: Changes in metabolite profiles caused by genetically determined obesity in mice
Source: Metabolomics. 2013 Oct 19;10(3):461–72. doi: 10.1007/s11306-013-0590-1 (PMC3984667; doi:10.1007/s11306-013-0590-1)
Supplement: Supplementary file 1 — Supplementary material 1 (DOCX 25 kb) [file 11306_2013_590_MOESM1_ESM.docx]

**Supplemental Table S1:** List of targeted metabolites using the Absolute*IDQ*^TM^ p150 Kit.

| Analyte | Short name | Biochemical name |
| --- | --- | --- |
| Acylcarnitines | C0 | Carnitine |
|  | C2 | Acetylcarnitine |
|  | C3 | Propionylcarnitine |
|  | C3:1 | Propenoylcarnitine |
|  | C3-OH | Hydroxypropionylcarnitine |
|  | C4 | Butyrylcarnitine |
|  | C4:1 | Butenylcarnitine |
|  | C4-OH (C3-DC) | Hydroxybutyrylcarnitine |
|  | C5 | Valerylcarnitine |
|  | C5:1 | Tiglylcarnitine |
|  | C5:1-DC | Glutaconylcarnitine |
|  | C5-DC (C6-OH) | Glutarylcarnitine (Hydroxyhexanoylcarnitine) |
|  | C5-M-DC | Methylglutarylcarnitine |
|  | C5-OH (C3-DC-M) | Hydroxyvalerylcarnitine (Methylmalonylcarnitine) |
|  | C6 (C4:1-DC) | Hexanoylcarnitine (Fumarylcarnitine) |
|  | C6:1 | Hexenoylcarnitine |
|  | C7-DC | Pimelylcarnitine |
|  | C8 | Octanoylcarnitine |
|  | C8:1 | Octenoylcarnitine |
|  | C9 | Nonaylcarnitine |
|  | C10 | Decanoylcarnitine |
|  | C10:1 | Decenoylcarnitine |
|  | C10:2 | Decadienylcarnitine |
|  | C12 | Dodecanoylcarnitine |
|  | C12:1 | Dodecenoylcarnitine |
|  | C12-DC | Dodecanedioylcarnitine |
|  | C14 | Tetradecanoylcarnitine |
|  | C14:1 | Tetradecenoylcarnitine |
|  | C14:1-OH | Hydroxytetradecenoylcarnitine |
|  | C14:2 | Tetradecadienylcarnitine |
|  | C14:2-OH | Hydroxytetradecadienylcarnitine |
|  | C16 | Hexadecanoylcarnitine |
|  | C16:1 | Hexadecenoylcarnitine |
|  | C16:1-OH | Hydroxyhexadecenoylcarnitine |
|  | C16:2 | Hexadecadienylcarnitine |
|  | C16:2-OH | Hydroxyhexadecadienylcarnitine |
|  | C16-OH | Hydroxyhexadecanoylcarnitine |
|  | C18 | Octadecanoylcarnitine |
|  | C18:1 | Octadecenoylcarnitine |
|  | C18:1-OH | Hydroxyoctadecenoylcarnitine |
|  | C18:2 | Octadecadienylcarnitine |
| Amino Acids | Arg-PTC | Arginine |
|  | Gln-PTC | Glutamine |
|  | Gly-PTC | Glycine |
|  | His-PTC | Histidine |
|  | Met-PTC | Methionine |
|  | Orn-PTC | Ornithine |
|  | Phe-PTC | Phenylalanine |
|  | Pro-PTC | Proline |
|  | Ser-PTC | Serine |
|  | Thr-PTC | Threonine |
|  | Trp-PTC | Tryptophan |
|  | Tyr-PTC | Tyrosine |
|  | Val-PTC | Valine |
|  | xLeu-PTC | Leucine / Isoleucine |
| Sugar | H1 | Hexose |
| Glycerophospholipids | lysoPC a C6:0 | lysoPhosphatidylcholine acyl C6:0 |
|  | lysoPC a C14:0 | lysoPhosphatidylcholine acyl C14:0 |
|  | lysoPC a C16:0 | lysoPhosphatidylcholine acyl C16:0 |
|  | lysoPC a C16:1 | lysoPhosphatidylcholine acyl C16:1 |
|  | lysoPC a C17:0 | lysoPhosphatidylcholine acyl C17:0 |
|  | lysoPC a C18:0 | lysoPhosphatidylcholine acyl C18:0 |
|  | lysoPC a C18:1 | lysoPhosphatidylcholine acyl C18:1 |
|  | lysoPC a C18:2 | lysoPhosphatidylcholine acyl C18:2 |
|  | lysoPC a C20:3 | lysoPhosphatidylcholine acyl C20:3 |
|  | lysoPC a C20:4 | lysoPhosphatidylcholine acyl C20:4 |
|  | lysoPC a C24:0 | lysoPhosphatidylcholine acyl C24:0 |
|  | lysoPC a C26:0 | lysoPhosphatidylcholine acyl C26:0 |
|  | lysoPC a C26:1 | lysoPhosphatidylcholine acyl C26:1 |
|  | lysoPC a C28:0 | lysoPhosphatidylcholine acyl C28:0 |
|  | lysoPC a C28:1 | lysoPhosphatidylcholine acyl C28:1 |
|  | PC aa C24:0 | Phosphatidylcholine diacyl C24:0 |
|  | PC aa C26:0 | Phosphatidylcholine diacyl C26:0 |
|  | PC aa C28:1 | Phosphatidylcholine diacyl C28:1 |
|  | PC aa C30:0 | Phosphatidylcholine diacyl C30:0 |
|  | PC aa C30:2 | Phosphatidylcholine diacyl C30:2 |
|  | PC aa C32:0 | Phosphatidylcholine diacyl C32:0 |
|  | PC aa C32:1 | Phosphatidylcholine diacyl C32:1 |
|  | PC aa C32:2 | Phosphatidylcholine diacyl C32:2 |
|  | PC aa C32:3 | Phosphatidylcholine diacyl C32:3 |
|  | PC aa C34:1 | Phosphatidylcholine diacyl C34:1 |
|  | PC aa C34:2 | Phosphatidylcholine diacyl C34:2 |
|  | PC aa C34:3 | Phosphatidylcholine diacyl C34:3 |
|  | PC aa C34:4 | Phosphatidylcholine diacyl C34:4 |
|  | PC aa C36:0 | Phosphatidylcholine diacyl C36:0 |
|  | PC aa C36:1 | Phosphatidylcholine diacyl C36:1 |
|  | PC aa C36:2 | Phosphatidylcholine diacyl C36:2 |
|  | PC aa C36:3 | Phosphatidylcholine diacyl C36:3 |
|  | PC aa C36:4 | Phosphatidylcholine diacyl C36:4 |
|  | PC aa C36:5 | Phosphatidylcholine diacyl C36:5 |
|  | PC aa C36:6 | Phosphatidylcholine diacyl C36:6 |
|  | PC aa C38:0 | Phosphatidylcholine diacyl C38:0 |
|  | PC aa C38:1 | Phosphatidylcholine diacyl C38:1 |
|  | PC aa C38:3 | Phosphatidylcholine diacyl C38:3 |
|  | PC aa C38:4 | Phosphatidylcholine diacyl C38:4 |
|  | PC aa C38:5 | Phosphatidylcholine diacyl C38:5 |
|  | PC aa C38:6 | Phosphatidylcholine diacyl C38:6 |
|  | PC aa C40:1 | Phosphatidylcholine diacyl C40:1 |
|  | PC aa C40:2 | Phosphatidylcholine diacyl C40:2 |
|  | PC aa C40:3 | Phosphatidylcholine diacyl C40:3 |
|  | PC aa C40:4 | Phosphatidylcholine diacyl C40:4 |
|  | PC aa C40:5 | Phosphatidylcholine diacyl C40:5 |
|  | PC aa C40:6 | Phosphatidylcholine diacyl C40:6 |
|  | PC aa C42:0 | Phosphatidylcholine diacyl C42:0 |
|  | PC aa C42:1 | Phosphatidylcholine diacyl C42:1 |
|  | PC aa C42:2 | Phosphatidylcholine diacyl C42:2 |
|  | PC aa C42:4 | Phosphatidylcholine diacyl C42:4 |
|  | PC aa C42:5 | Phosphatidylcholine diacyl C42:5 |
|  | PC aa C42:6 | Phosphatidylcholine diacyl C42:6 |
|  | PC ae C30:0 | Phosphatidylcholine acyl-alkyl C30:0 |
|  | PC ae C30:1 | Phosphatidylcholine acyl-alkyl C30:1 |
|  | PC ae C30:2 | Phosphatidylcholine acyl-alkyl C30:2 |
|  | PC ae C32:1 | Phosphatidylcholine acyl-alkyl C32:1 |
|  | PC ae C32:2 | Phosphatidylcholine acyl-alkyl C32:2 |
|  | PC ae C34:0 | Phosphatidylcholine acyl-alkyl C34:0 |
|  | PC ae C34:1 | Phosphatidylcholine acyl-alkyl C34:1 |
|  | PC ae C34:2 | Phosphatidylcholine acyl-alkyl C34:2 |
|  | PC ae C34:3 | Phosphatidylcholine acyl-alkyl C34:3 |
|  | PC ae C36:0 | Phosphatidylcholine acyl-alkyl C36:0 |
|  | PC ae C36:1 | Phosphatidylcholine acyl-alkyl C36:1 |
|  | PC ae C36:2 | Phosphatidylcholine acyl-alkyl C36:2 |
|  | PC ae C36:3 | Phosphatidylcholine acyl-alkyl C36:3 |
|  | PC ae C36:4 | Phosphatidylcholine acyl-alkyl C36:4 |
|  | PC ae C36:5 | Phosphatidylcholine acyl-alkyl C36:5 |
|  | PC ae C38:0 | Phosphatidylcholine acyl-alkyl C38:0 |
|  | PC ae C38:1 | Phosphatidylcholine acyl-alkyl C38:1 |
|  | PC ae C38:2 | Phosphatidylcholine acyl-alkyl C38:2 |
|  | PC ae C38:3 | Phosphatidylcholine acyl-alkyl C38:3 |
|  | PC ae C38:4 | Phosphatidylcholine acyl-alkyl C38:4 |
|  | PC ae C38:5 | Phosphatidylcholine acyl-alkyl C38:5 |
|  | PC ae C38:6 | Phosphatidylcholine acyl-alkyl C38:6 |
|  | PC ae C40:0 | Phosphatidylcholine acyl-alkyl C40:0 |
|  | PC ae C40:1 | Phosphatidylcholine acyl-alkyl C40:1 |
|  | PC ae C40:2 | Phosphatidylcholine acyl-alkyl C40:2 |
|  | PC ae C40:3 | Phosphatidylcholine acyl-alkyl C40:3 |
|  | PC ae C40:4 | Phosphatidylcholine acyl-alkyl C40:4 |
|  | PC ae C40:5 | Phosphatidylcholine acyl-alkyl C40:5 |
|  | PC ae C40:6 | Phosphatidylcholine acyl-alkyl C40:6 |
|  | PC ae C42:0 | Phosphatidylcholine acyl-alkyl C42:0 |
|  | PC ae C42:1 | Phosphatidylcholine acyl-alkyl C42:1 |
|  | PC ae C42:2 | Phosphatidylcholine acyl-alkyl C42:2 |
|  | PC ae C42:3 | Phosphatidylcholine acyl-alkyl C42:3 |
|  | PC ae C42:4 | Phosphatidylcholine acyl-alkyl C42:4 |
|  | PC ae C42:5 | Phosphatidylcholine acyl-alkyl C42:5 |
|  | PC ae C44:3 | Phosphatidylcholine acyl-alkyl C44:3 |
|  | PC ae C44:4 | Phosphatidylcholine acyl-alkyl C44:4 |
|  | PC ae C44:5 | Phosphatidylcholine acyl-alkyl C44:5 |
|  | PC ae C44:6 | Phosphatidylcholine acyl-alkyl C44:6 |
| Sphingolipids | SM (OH) C14:1 | Hydroxysphingomyeline C14:1 |
|  | SM C16:0 | Sphingomyeline C16:0 |
|  | SM C16:1 | Sphingomyeline C16:1 |
|  | SM (OH) C16:1 | Hydroxysphingomyeline C16:1 |
|  | SM C18:0 | Sphingomyeline C18:0 |
|  | SM C18:1 | Sphingomyeline C18:1 |
|  | SM C20:2 | Sphingomyeline C20:2 |
|  | SM C22:3 | Sphingomyeline C22:3 |
|  | SM (OH) C22:1 | Hydroxysphingomyeline C22:1 |
|  | SM (OH) C22:2 | Hydroxysphingomyeline C22:2 |
|  | SM C24:0 | Sphingomyeline C24:0 |
|  | SM C24:1 | Sphingomyeline C24:1 |
|  | SM (OH) C24:1 | Hydroxysphingomyeline C24:1 |
|  | SM C26:0 | Sphingomyeline C26:0 |
|  | SM C26:1 | Sphingomyeline C26:1 |
