# Supplementary material for: Changes in metabolite profiles caused by genetically determined obesity in mice
Source: Metabolomics. 2013 Oct 19;10(3):461–72. doi: 10.1007/s11306-013-0590-1 (PMC3984667; doi:10.1007/s11306-013-0590-1)
Supplement: Supplementary file 2 — Supplementary material 2 (DOCX 23 kb) [file 11306_2013_590_MOESM2_ESM.docx]

**Supplemental Table S2** Sex, diet and line effects on metabolites levels of male and female BFMI, B6 and F_1_ mice on SMD and HFD.

| Metabolites short name | Metabolite biochemical name | BFMI | | | | |  | B6 | | | | |  | F_1_ | | | | |  | *P* values of the effects of | | |  | *P* values of pairwise comparisons of line effects | | |
| --- | --- | --- | --- | --- | --- | --- | --- | --- | --- | --- | --- | --- | --- | --- | --- | --- | --- | --- | --- | --- | --- | --- | --- | --- | --- | --- |
|  |  |  |  |  |  |  |  |  |  |  |  |  |  |  |  |  |  |  |  |  |  |  |  |  |  |  |
|  |  | male | |  | female | |  | male | |  | female | |  | male | |  | female | |  | Sex | Diet | Line |  | BFMI/B6 | BFMI/F_1_ | B6/F_1_ |
|  |  | SMD | HFD |  | SMD | HFD |  | SMD | HFD |  | SMD | HFD |  | SMD | HFD |  | SMD | HFD |  |  |  |  |  |  |  |  |
| PC aa C36:3 | Phosphatidylcholine diacyl C36:3 | 168.6 | 147.6 |  | 118.5 | 110.6 |  | 176.1 | 155.2 |  | 175.5 | 188.5 |  | 197.2 | 211.4 |  | 152.5 | 194.8 |  | n.s. | n.s. | 0.032 |  | n.s. | 0.037 | n.s. |
|  |  | (54.5) | (69.5) |  | (35.1) | (51.6) |  | (88.8) | (40.1) |  | (72.2) | (28.6) |  | (102.5) | (95.8) |  | (89.3) | (68.9) |  |  |  |  |  |  |  |  |
| PC aa C40:2 | Phosphatidylcholine diacyl C40:2 | 0.59 | 0.49 |  | 0.46 | 0.37 |  | 0.55 | 0.54 |  | 0.6 | 0.6 |  | 0.67 | 0.65 |  | 0.59 | 0.6 |  | n.s. | n.s. | 0.011 |  | n.s. | 0.008 | n.s. |
|  |  | (0.2) | (0.16) |  | (0.09) | (0.08) |  | (0.2) | (0.2) |  | (0.2) | (0.2) |  | (0.28) | (0.23) |  | (0.2) | (0.16) |  |  |  |  |  |  |  |  |
| PC aa C40:3 | Phosphatidylcholine diacyl C40:3 | 1.1 | 0.78 |  | 0.67 | 0.53 |  | 0.9 | 0.9 |  | 0.9 | 1 |  | 1.1 | 1.1 |  | 0.99 | 1.08 |  | n.s. | n.s. | 0.046 |  | n.s. | 0.04 | n.s. |
|  |  | (0.66) | (0.35) |  | (0.32) | (0.29) |  | (0.5) | (0.4) |  | (0.6) | (0.2) |  | (0.75) | (0.54) |  | (0.57) | (0.34) |  |  |  |  |  |  |  |  |
| PC aa C40:4 | Phosphatidylcholine diacyl C40:4 | 4.2 | 3.6 |  | 2.8 | 2.5 |  | 4.3 | 4 |  | 3.8 | 4.5 |  | 5.1 | 5.3 |  | 4.3 | 4.9 |  | n.s. | n.s. | 0.042 |  | n.s. | 0.04 | n.s. |
|  |  | (2.1) | (2.1) |  | (1.4) | (1) |  | (2.7) | (1.6) |  | (1.8) | (1.4) |  | (3.8) | (2.9) |  | (2.6) | (1.8) |  |  |  |  |  |  |  |  |
| PC aa C42:1 | Phosphatidylcholine diacyl C42:1 | 0.16 | 0.16 |  | 0.16 | 0.13 |  | 0.18 | 0.16 |  | 0.17 | 0.17 |  | 0.15 | 0.17 |  | 0.21 | 0.16 |  | n.s. | n.s. | 0.005 |  | 0.011 | 0.014 | n.s. |
|  |  | (0.02) | (0.02) |  | (0.08) | (0.01) |  | (0.03) | (0.02) |  | (0.03) | (0.06) |  | (0.01) | (0.03) |  | (0.05) | (0.02) |  |  |  |  |  |  |  |  |
| PC aa C42:2 | Phosphatidylcholine diacyl C42:2 | 0.18 | 0.21 |  | 0.18 | 0.16 |  | 0.19 | 0.15 |  | 0.2 | 0.22 |  | 0.21 | 0.18 |  | 0.23 | 0.16 |  | n.s. | 0.045 | n.s. |  | n.s. | n.s. | n.s. |
|  |  | (0.01) | (0.04) |  | (0.07) | (0.02) |  | (0.03) | (0.02) |  | (0.04) | (0.12) |  | (0.02) | (0.01) |  | (0.06) | (0.01) |  |  |  |  |  |  |  |  |
| PC aa C42:5 | Phosphatidylcholine diacyl C42:5 | 0.42 | 0.39 |  | 0.32 | 0.25 |  | 0.37 | 0.4 |  | 0.4 | 0.43 |  | 0.42 | 0.44 |  | 0.43 | 0.37 |  | n.s. | n.s. | 0.019 |  | n.s. | 0.016 | n.s. |
|  |  | (0.15) | (0.11) |  | (0.04) | (0.08) |  | (0.07) | (0.1) |  | (0.1) | (0.16) |  | (0.12) | (0.14) |  | (0.12) | (0.05) |  |  |  |  |  |  |  |  |
| PC ae C42:1 | Phosphatidylcholine acyl-alkyl C42:1 | 0.77 | 0.85 |  | 0.63 | 0.63 |  | 0.9 | 0.8 |  | 1 | 1.21 |  | 0.98 | 0.84 |  | 0.79 | 0.85 |  | n.s. | n.s. | 0.015 |  | 0.02 | 0.048 | n.s |
|  |  | (0.12) | (0.18) |  | (0.15) | (0.2) |  | (0.2) | (0.2) |  | (0.34) | (0.85) |  | (0.26) | (0.13) |  | (0.35) | (0.19) |  |  |  |  |  |  |  |  |
| PC ae C44:3 | Phosphatidylcholine acyl-alkyl C44:3 | 0.14 | 0.15 |  | 0.13 | 0.1 |  | 0.16 | 0.13 |  | 0.16 | 0.2 |  | 0.15 | 0.15 |  | 0.16 | 0.17 |  | n.s. | n.s. | 0.007 |  | 0.017 | 0.014 | n.s |
|  |  | (0.03) | (0.03) |  | (0.03) | (0.01) |  | (0.04) | (0.03) |  | (0.03) | (0.07) |  | (0.05) | (0.02) |  | (0.05) | (0.05) |  |  |  |  |  |  |  |  |
| PC ae C44:4 | Phosphatidylcholine acyl-alkyl C44:4 | 0.15 | 0.18 |  | 0.18 | 0.12 |  | 0.18 | 0.15 |  | 0.18 | 0.19 |  | 0.18 | 0.17 |  | 0.22 | 0.18 |  | n.s. | n.s. | 0.002 |  | n.s. | 0.012 | n.s. |
|  |  | (0.03) | (0.04) |  | (0.13) | (0.04) |  | (0.07) | (0.03) |  | (0.06) | (0.09) |  | (0.06) | (0.05) |  | (0.04) | (0.05) |  |  |  |  |  |  |  |  |
| C14 | Tetradecanoylcarnitine | 0.08 | 0.08 |  | 0.08 | 0.08 |  | 0.09 | 0.1 |  | 0.09 | 0.1 |  | 0.09 | 0.15 |  | 0.11 | 0.11 |  | n.s. | n.s. | 0.006 |  | n.s. | 0.005 | n.s. |
|  |  | (0.03) | (0.01) |  | (0.02) | (0.01) |  | (0.02) | (0.02) |  | (0.02) | (0.01) |  | (0.02) | (0.12) |  | (0.07) | (0.01) |  |  |  |  |  |  |  |  |
| C14:1 | Tetradecanoylcarnitine | 0.13 | 0.12 |  | 0.13 | 0.1 |  | 0.13 | 0.14 |  | 0.13 | 0.14 |  | 0.14 | 0.16 |  | 0.13 | 0.14 |  | n.s. | n.s. | 0.006 |  | 0.042 | 0.006 | n.s. |
|  |  | (0.03) | (0.02) |  | (0.03) | (0.02) |  | (0.03) | (0.02) |  | (0.02) | (0.03) |  | (0.05) | (0.05) |  | (0.02) | (0.02) |  |  |  |  |  |  |  |  |
| C18:1 | Octadecanoylcarnitine | 0.15 | 0.16 |  | 0.17 | 0.13 |  | 0.17 | 0.21 |  | 0.2 | 0.22 |  | 0.18 | 0.27 |  | 0.21 | 0.22 |  | n.s. | n.s. | 0.004 |  | 0.021 | 0.006 | n.s. |
|  |  | (0.05) | (0.04) |  | (0.06) | (0.04) |  | (0.06) | (0.09) |  | (0.04) | (0.03) |  | (0.03) | (0.16) |  | (0.13) | (0.06) |  |  |  |  |  |  |  |  |
| Ser | Serine | 114.5 | 114.8 |  | 68.4 | 136 |  | 74.7 | 80 |  | 80.1 | 110.5 |  | 83.2 | 76.7 |  | 80.8 | 72.8 |  | n.s. | n.s. | 0.028 |  | n.s. | 0.043 | n.s. |
|  |  | (13.1) | (47.7) |  | (11.6) | (70.8) |  | (15.4) | (18.6) |  | (28.6) | (82.8) |  | (13.8) | (16.3) |  | (29.3) | (25.1) |  |  |  |  |  |  |  |  |

Only those metabolites are listed showing significant differences between sex, diet, or lines. Values are shown as means and standard deviation in parenthesis. Pairwise comparisons were corrected for multiple testing using Bonferroni correction. Differences were considered statistically significant at *p* < 0.05 (n.s., not significant).
