# Supplementary material for: Changes in metabolite profiles caused by genetically determined obesity in mice
Source: Metabolomics. 2013 Oct 19;10(3):461–72. doi: 10.1007/s11306-013-0590-1 (PMC3984667; doi:10.1007/s11306-013-0590-1)
Supplement: Supplementary file 3 — Supplementary material 3 (DOCX 28 kb) [file 11306_2013_590_MOESM3_ESM.docx]

**Supplemental Table S3** Variations in the *Ccna2* promoter and the resultant effect in BFMI mice compared with B6 (reference) mice.

| Variation-ID | Position | B6 Allele | BFMI Allele | Genomic region  (Exosc9 / Ccna2) | rsID | Function | Factor | Effect in BFMI relative to B6 | Score | Expression of TF or miRNA in different tissues | | | |
| --- | --- | --- | --- | --- | --- | --- | --- | --- | --- | --- | --- | --- | --- |
|  |  |  |  |  |  |  |  |  |  | Liver | WAT | BAT | Array-Mean |
| SNP1 | 36471433 | G | A | Promoter | rs49416676 | TF-regulation | RORA  c-FOS | gain  silencing | 8.96  7.63 🡺 11.25 | 972.02  105.82 | 197.69  110.49 | 335.44  431.88 | 251.1  3409.4 |
| SNP2 | 36471338 | G | A | Promoter | rs50741947 | TF-regulation | NKX (NKX2-3)  SOX17  SOX9  FREAC7 (FOXL1)  SPI-B  FREAC3 (FOXC1)  FREAC2 (FOXF2)  SRY  SOX5  FREAC4 (FOXD1) | gain  gain  gain  gain  loss  loss  enhancing  enhancing  enhancing  enhancing | 5.19  8.99  9.68  5.23  5.36  5.24  14.87 🡺 12.47  11.66 🡺 8.05  10.90 🡺 6.62  12.34 🡺 11.36 | 5.39  6.73  40.12  4.64  4.68  10.94  6.52  4.64  109.43  4.64 | 4.64  33.44  171.12  4.64  6.28  30.28  5.64  4.64  12.00  4.64 | 4.64  47.88  12.21  4.64  4.68  10.37  5.97  4.70  22.25  4.64 | 4.81  27.8  80.9  4.78  367.4  168.9  10.9  6.29  64.5  35.5 |
| SNP3 | 36471316 | A | G | Promoter | rs46760941 | TF-regulation | FREAC7 (FOXL1)  GATA2  GATA3  MZF_1-4 (MZF1)  Gklf  SPI-B | loss  loss  loss  loss  silencing  silencing | 6.68  3.36  3.97  4.92  5.65 🡺 6.67  4.67 🡺 5.76 | 4.64  13.99  90.6  4.69  41.00  4.68 | 4.64  17.46  134.99  4.69  669.04  6.28 | 4.64  29.31  4.79  4.66  1223.1  4.68 | 4.78  160.4  4.95  5.27  1911.4  367.4 |
| SNP4 | 36471291 | A | G | Promoter | rs50888702 | TF-regulation | Irf-1  SPI-1  SPI-B  GATA-3  Gklf | loss  loss  loss  enhancing  silencing | 10.24  4.72  6.32  5.29 🡺 4.09  8.04 🡺 10.44 | 339.85  12.00  4.68  4.95  41.00 | 259.75  13.43  6.28  13.99  669.04 | 301.87  19.35  4.68  4.79  1223.1 | 698.3  447.8  367.4  90.6  1911.4 |
| SNP5 | 36471264 | G | A | Promoter | rs48075421 | TF-regulation | Gklf  SP1  Pax-2  TFAP2A | gain  loss  enhancing  silencing | 5.14  6.50  4.85 🡺 4.54  4.48 🡺 4.71 | 41.00  780.3  173.0  7.20 | 669.04  1694.2  126.4  9.04 | 1223.1  1048.8  126.4  4.85 | 1911.4  2387.5  136.4  7.60 |
| SNP6 | 36471206 | A | G | Promoter | rs45964814 | TF-regulation | TFAP2A  MZF1_5-13 (MZF1)  MZF1_1-4 (MZF1)  Gklf (Klf4)  SPI-1  SPI-B | gain  gain  gain  enhancing  enhancing  enhancing | 4.53  6.28  7.94  9.50 🡺 8.44  8.58 🡺 5.50  8.91🡺 5.21 | 7.20  4.69  4.69  41.00  12.00  4.68 | 9.04  4.69  4.69  669.04  13.43  6.28 | 4.85  4.66  4.66  1223.1  19.35  4.68 | 7.60  5.27  5.27  1911.4  447.8  367.4 |
| SNP7 | 36471126 | C | G | Promoter | rs51039343 | TF-regulation | Elk-1  SP1  TFAP2A  SPI-1 | loss  enhancing  enhancing  unchanged | 5.78  7.61 🡺 6.14  7.25 🡺6.66  4.25 🡺 4.38 | 4.64  780.3  7.20  12.00 | 4.64  1694.2  9.04  13.43 | 4.64  1048.8  4.85  19.35 | 8.00  2387.5  7.60  447.8 |
| DEL1 | 36471074 | A | - | Promoter | novel | TF-regulation | Hen1 (Nhlh1)  Myf (Myog)  Elk-1  TFAP2A  SPI-1 | gain  gain  loss  loss  silencing | 8.47  8.18  7.25  6.99  4.38 🡺 5.25 | 4.64  4.64  4.64  7.20  12.00 | 4.64  4.64  4.64  9.04  13.43 | 4.64  6.11  4.64  4.85  19.35 | 4.68  10.94  8.00  7.60  447.8 |
| SNP8 | 36471071 | G | T | 5'UTR / Exon1 | novel | TF-regulation | identical to DEL1 | | | | | |  |
| SNP9 | 36470786 | T | C | 5'UTR / Exon1 | novel | TF-regulation | TFAP2A  SP1 | gain  loss | 5.31  7.33 | 7.20  780.3 | 9.04  1694.2 | 4.85  1048.8 | 7.60  2387.5 |
| SNP10 | 36470691 | C | T | Exon1 / coding | novel | synonymous coding | YTG | Leucin | - | - | - | - | - |
| SNP11 | 36467695 | G | A | Exon4 / coding | rs49497566 | synonymous coding | CAR | Glutamin | - | - | - | - | - |
| SNP12 | 36465032 | T | C | Exon8 / 3'UTR | rs29940755 | unknown | - | - | - | - | - | - | - |
| SNP13 | 36464877 | A | G | Exon8 / 3'UTR | rs29939979 | miRNA-regulation | mmu-miR-539 | gain | -11.5 | down | down | down | - |
| SNP14 | 36464857 | G | A | Exon8 / 3'UTR | rs29939976 | miRNA-regulation | mmu-miR-669 | gain | -12.8 | down | down- | down | - |
| SNP15 | 36464725 | C | T | Exon8 / 3'UTR | rs45880100 | unknown | - | - |  | - | - | - | - |
| INS1 | 36464688 | - | T | Exon8 / 3'UTR | rs29939053 | miRNA-regulation | mmu-miR-671 | gain | -10.0 | down | n.a. | n.a. | - |
| SNP16 | 36464653 | G | C | Exon8 / 3'UTR | rs29939050 | unknown | - | - |  | - | - | - | - |
| SNP17 | 36464611 | A | T | Exon8 / 3'UTR | rs29939047 | miRNA-regulation | mmu-miR-342  mmu-miR-705  mmu-miR-450  mmu-miR-762  mmu-miR-296 | gain  gain  gain  gain  gain | -10.8  -12.8  -12.5  -12.2  -11.7 | n.a.  n.a.  n.a.  n.a.  n.a. | n.a.  n.a.  n.a.  n.a.  n.a. | n.a.  n.a.  n.a.  n.a.  n.a. | -  -  -  -  - |
| DEL2 | 36464525 | ACAA | - | Exon8 / 3'UTR | novel | miRNA-regulation | mmu-miR-208 | loss | -11.4 | up | n.a. | n.a. | - |
| SNP18 | 36464511 | A | G | Exon8 / 3'UTR | rs29939044 | - | - | - | - | -. | - | - | - |
| SNP19 | 36464492 | C | T | Exon8 / 3'UTR | rs29938061 | miRNA-regulation | mmu-miR-466 | gain | < -10.0 | down | up | n.a. | - |
| SNP20 | 36464477 | A | G | Exon8 / 3'UTR | novel | miRNA-regulation | mmu-miR-466  mmu-miR-574  mmu-miR-362  mmu-miR-342  mmu-miR-467 | gain  gain  gain  gain  gain | < -15.0  -17.2  -10.4  -11.1  -17.8 | down  n.a.  n.a.  n.a.  n.a. | up  n.a.  up  n.a.  n.a. | n.a.  n.a.  up  n.a.  n.a. | -  -  -  - |
| INS2 | 36464469 | - | G | Exon8 / 3'UTR | novel | miRNA-regulation | mmu-miR-466  mmu-miR-467  mmu-miR-297  mmu-miR-669  mmu-miR-297  mmu-miR-669 | gain  gain  gain  gain  loss  loss | < -15.0  < -15.0  < -13.0  < -15.0  < -10.0  < -12.00 | down  n.a.  n.a.  down  n.a. down | up  n.a.  n.a.  down  n.a. down | n.a.  n.a.  n.a.  down  n.a. down | -  -  -  -  - |
| INS3 | 36464464 | - | GTGTATATACATACACACACATATACAC | Exon8 / 3'UTR | novel | miRNA-regulation | mmu-miR-466  mmu-miR-467  mmu-miR-297  mmu-miR-466  mmu-miR-669f | enhancing  enhancing  enhancing  gain  loss | < -20.0 🡺< -18.0  < -18.0 🡺 <-16.0  -20.1🡺-14.3  < -12.0  < -12.0 | down  n.a.  n.a.  down  down | up  n.a.  n.a.  up  down | n.a.  n.a.  n.a.  n.a.  down | -  -  -  -  - |
| SNP21 | 36464447 | G | C | Exon8 / 3'UTR | rs47451785 | - |  |  |  |  |  |  |  |
| SNP22 | 36464390 | G | C | Exon8 / 3'UTR | rs29938058 | miRNA-regulation | mmu-miR-470  mmu-miR-330  mmu-miR-362  mmu-miR-211  mmu-miR-204 | loss  loss  loss  loss  loss | -17.4  -15.2  -11.9  -11.8  -10.6 | n.a.  n.a.  n.a.  n.a.  n.a. | n.a.  n.a.  up  up  up | n.a.  n.a.  up  up  up | -  -  -  -  - |
| SNP23 | 36464274 | C | T | Exon8 / 3'UTR | rs29938055 | - | - | - | - | - | - | - | - |
| SNP24 | 36463910 | A | G | Exon8 / 3'UTR | rs29944107 | miRNA-regulation | mmu-miR-759  mmu-miR-665  mmu-miR-433 | gain  gain  gain | -10.5  -17.6  -10.1 | n.a.  n.a.  n.a. | n..a.  down  n.a. | n.a.  down  n.a. | -  -  - |

Transcription factor binding sites were determined with the web tool CONSITE (Sandelin et al*.*, 2004) using human and mouse transcription factor model matrices and a scoring threshold of 80%. The 3’ UTR of the *Ccna2* reference transcript (NM_009828) was scanned for binding sites of known mouse miRNAs using the web-programme PITA (Kertesz et al., 2007) using standard parameter. Resulting energetic scores estimate the free binding energy in the seed region of the miRNA-mRNA duplex and thus the binding strength of the miRNA to the given 3’UTR site. Only scores equal or below -10 were considered as these are likely to be functional in endogenous miRNA expression levels ([Kertesz et al., 2007](#_ENREF_16)). Expression data of transcription factors were taken from the arrays GeneAtlas MOE430 and GNF1M via the web tool BioGPS (http://biogps.org). MiRNA expression data was obtained from the Gene Expression Atlas (http://www.ebi.ac.uk/gxa/).
