# Supplementary material for: Changes in metabolite profiles caused by genetically determined obesity in mice
Source: Metabolomics. 2013 Oct 19;10(3):461–72. doi: 10.1007/s11306-013-0590-1 (PMC3984667; doi:10.1007/s11306-013-0590-1)
Supplement: Supplementary file 4 — Supplementary material 4 (DOCX 16 kb) [file 11306_2013_590_MOESM4_ESM.docx]

**Supplemental Table S4:** Primer pairs for gene expression and sequencing studies in target genes.

| Gene | Forward primer (5’ → 3’) | Reverse primer (5’ → 3’) | Temp. | Template | Analysis |
| --- | --- | --- | --- | --- | --- |
| *Chka* | GCCATTCTTGCAGAGAGGTC | AATGTGGCCATTTTCTCAGC | 60°C | cDNA | qPCR |
| *Ccna2* | GAGAATGTCAACCCCGAAAA | GCAGTGACATGCTCATCGTT | 60°C | cDNA | qPCR |
| *Trpc3* | TGGATTGCACCTTGTAGCAG | ACGTGAACTGGGTGGTCTTC | 60°C | cDNA | qPCR |
| *Pla2g1b* | GGAGTGATCCCCTGAAGGAT | TGAAGTCCTCGCATTTCTGGT | 60°C | cDNA | qPCR |
| *b-actin* | GACGGCCAGGTCACACTAT | CTTCTGCATCCTGTCAGCAA | 60°C | cDNA | qPCR |
| *Rps25* | TCGACAAAGCGACATACGAC | CCACCCTTTGTGTTTCTGGT | 60°C | cDNA | qPCR |
| *Ccna2-1* | GTGGGGGTGGGGTAGTTTAC | CGGCTGCTGGTCTTGTAGTTC | 61°C | gDNA | Sequencing |
| *Ccna2-2* | GCTCCCGCCCTGTAAGATT | TGACCCCGGGCATTTTT | 61°C | gDNA | Sequencing |
| *Ccna2-3* | CCCTGGGGCTAAAGTTGTGAATC | ACTTGGGAGGCTGAGGTAGGTG | 56°C | gDNA | Sequencing |
| *Ccna2-4* | AGCCTGCCTTCACCATTCAT | TCAGTTCTCCCAAAAACATTGC | 61°C | gDNA | Sequencing |
| *Ccna2-5* | GCATTTGGCTGTGAACTACATTG | TGGTGGGTTGAGAAGAGAAACA | 62°C | gDNA | Sequencing |
| *Ccna2-6* | TTCACCAGACCTACCTCAAAGC | ACCAGAAGGCACCAAGTAAAGG | 61°C | gDNA | Sequencing |
| *Ccna2-7* | ACCAAACCAAACTGCTGACTTG | GAGAAAAATGGGCAAACAAAGC | 62°C | gDNA | Sequencing |

The temperature (Temp.) indicates the annealing temperature of the PCR reaction.
